# Control of antiferromagnetic spin axis orientation in bilayer Fe/CuMnAs films

**DOI:** 10.1038/s41598-017-11653-8

**Published:** 2017-09-11

**Authors:** P. Wadley, K. W. Edmonds, M. R. Shahedkhah, R. P. Campion, B. L. Gallagher, J. Železný, J. Kuneš, V. Novák, T. Jungwirth, V. Saidl, P. Němec, F. Maccherozzi, S. S. Dhesi

**Affiliations:** 1 School of Physics and Astronomy, University of Nottingham, Nottingham, NG7 2RD United Kingdom; 2 Institute of Physics, Czech Academy of Sciences, Cukrovarnická 10, 162 00, Praha 6, Czech Republic; 3 Max Planck Institute for Chemical Physics of Solids, 01187 Dresden, Germany; 4 Institute of Physics, Czech Academy of Sciences, Na Slovance 1999/2, 182 21, Praha 8, Czech Republic; 5 Institute of Solid State Physics, TU Wien, Wiedner Hauptstr. 8, 1040 Wien, Austria; 6 Faculty of Mathematics and Physics, Charles University, Ke Karlovu 3, 121 16, Praha 2, Czech Republic; 7 Diamond Light Source, Chilton, Didcot, Oxfordshire, OX11 0DE United Kingdom

## Abstract

Using x-ray magnetic circular and linear dichroism techniques, we demonstrate a collinear exchange coupling between an epitaxial antiferromagnet, tetragonal CuMnAs, and an Fe surface layer. A small uncompensated Mn magnetic moment is observed which is antiparallel to the Fe magnetization. The staggered magnetization of the 5 nm thick CuMnAs layer is rotatable under small magnetic fields, due to the interlayer exchange coupling. This allows us to obtain the x-ray magnetic linear dichroism spectra for different crystalline orientations of CuMnAs in the (001) plane. This is a key parameter for enabling the understanding of domain structures in CuMnAs imaged using x-ray magnetic linear dichroism microscopy techniques.

## Introduction

**Figure 1:**
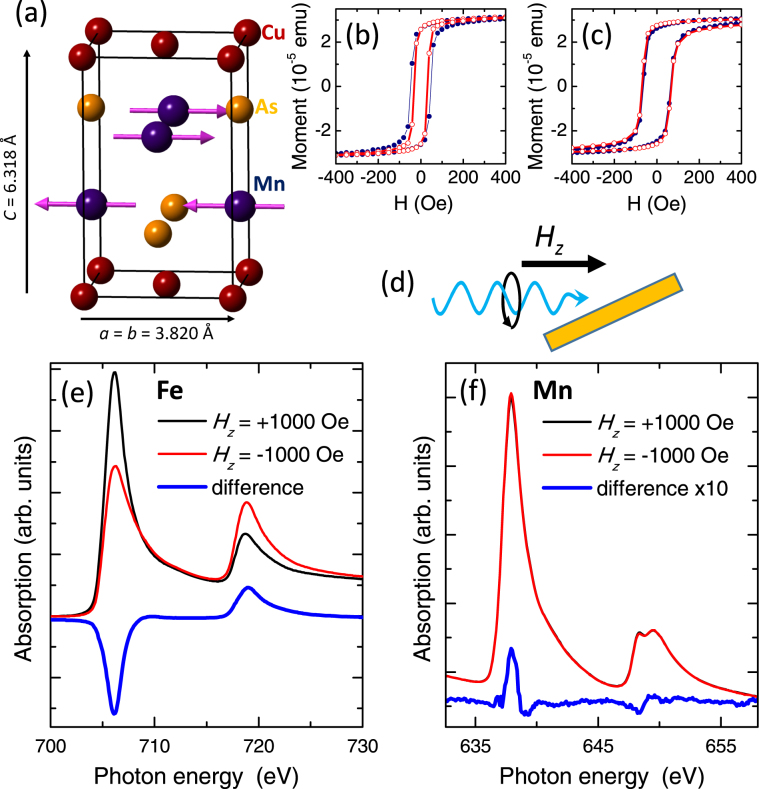
Crystal structure, magnetometry and XMCD. (**a**) Crystal structure of tetragonal CuMnAs. (**b**) and (**c**) SQUID hysteresis loops for the Fe/CuMnAs film at 200 K and 2 K respectively, for magnetic field along the substrate [110] (filled symbols) and [100] (open symbols) axes. (**d**) Experimental geometry for the XMCD measurements. (**e**) Fe *L*_2,3_ and (**f**) Mn *L*_2,3_ absorption spectra for magnetic fields applied parallel and antiparallel to the x-ray helicity vector, and the difference (XMCD) spectra, at sample temperature 250 K. The Mn XMCD is scaled by a factor of 10 for clarity.

Antiferromagnetic (AF) spintronics is an emerging field which aims to utilize the particular properties of AF materials for information storage and processing applications^1^. The collinear antiferromagnet tetragonal CuMnAs is of particular interest due to its crystal structure, in which the two Mn spin sublattices form inversion partners in a centrosymmetric lattice (Fig. 1a)^2^. Due to spin-orbit coupling, an electric current results in a local spin polarization, of opposite sign on each sublattice, which can induce a torque large enough to rotate the staggered magnetization between stable configurations^3–5^. Further, theoretical studies have predicted the presence of Dirac band crossings in both the tetragonal and orthorhombic phases of CuMnAs, co-existing with and influenced by the AF order^6, 7^. Methods to image and control the AF order in CuMnAs are therefore of substantial current interest. X-ray magnetic linear dichroism (XMLD) provides one of the few tools to measure AF order. XMLD photoemission electron microscopy (XMLD-PEEM) in particular is able to map out the AF contrast down to the nanometre scale. However, to determine the AF domain vectors from this data one needs to understand the angular dependence of the XMLD line shape in the given material. In this report we demonstrate the line shape of the XMLD in CuMnAs along different crystalline directions which will provide greater understanding of the domain structures in this important material.

The physics of exchange coupling at a ferromagnetic (FM)/antiferromagnetic (AF) interface has been widely studied, both for fundamental understanding and for applications in magnetic storage and memory technologies. Characteristic features of such interfaces include enhancement of the coercivity and a shift of the hysteresis loop (exchange bias) of the FM layer^8^. Studies of epitaxial interfaces between crystalline materials offer particular insights, due to their well-controlled interface structures and magnetocrystalline anisotropies^9^. The configuration of the spins in the AF layer – whether bulk or surface, fully anti-aligned or partially uncompensated, rotatable or frozen in place – can strongly affect the behaviour of the FM layer^10^. The AF configuration in FM/AF bilayers has been explored directly, using x-ray spectroscopy and spectromicroscopy techniques as well as tunnelling anisotropic magnetoresistance^11–14^. Such experiments have shown the close connection between the rotation and pinning of AF moments and the hysteresis of the FM layer. This also provides a means to manipulate the spins in an AF layer for potential spintronic applications^9, 15^.

Ab initio calculations indicate that the stable configurations of the staggered magnetization in tetragonal CuMnAs lie in the (001) plane, where a biaxial magnetic anisotropy is expected due to the crystal symmetry^2, 16^. However, the tetragonal polytype of CuMnAs is stabilized by growth on III-V substrates (GaP or GaAs), which leads to an in-plane uniaxial magnetic anisotropy^16, 17^. Similar anisotropies are commonly found in FM/III-V films, due to the broken symmetry of the III-V surface^18^.

Here we present a study of the magnetic coupling and XMLD spectra in a bilayer film consisting of FM Fe and AF CuMnAs. We combine XMLD as well as x-ray magnetic circular dichroism (XMCD) to obtain element specific information on the FM layer as well as both compensated and uncompensated magnetic moments in the AF layer. In crystalline materials, the XMLD in particular contains rich information on the atomic and magnetic structure. Crystalline anisotropy of XMLD spectra, in which the spectral lineshape depends strongly on the direction of the x-ray polarization vector with respect to the crystallographic axes, has been observed in theoretical and experimental studies of a wide variety of magnetic materials including metals^19, 20^, oxides^21–23^ and diluted magnetic semiconductors^24^. Here we utilize the exchange coupling between the Fe layer and rotatable AF CuMnAs spins to reveal the anisotropic XMLD spectra for tetragonal CuMnAs, which are compared to ab initio calculations.

## Methods and Results

### Growth, structure and magnetometry

The sample studied consists of a 2 nm Al/2 nm Fe/5 nm CuMnAs film grown on a GaP(001) substrate by molecular beam epitaxy. The substrate temperature during growth was 260 °C for the CuMnAs layer and 0 °C for the Fe layer and the protective Al cap. The layers were grown in the same ultra-high vacuum chamber, to ensure a clean interface between them. Previous studies have shown that tetragonal CuMnAs is lattice-matched to GaP(001) through a 45° rotation of the unit cell^2^. The measurements described below confirm the epitaxial relationship Fe(001) [110] || CuMnAs(001) [100] || GaP(001) [110]. Figure 1b and c show magnetization loops for the film measured by superconducting quantum interference device (SQUID) magnetometry along the in-plane [110] and [100] directions of the GaP substrate, at temperatures of 200 K and 2 K respectively. Negligible exchange bias is observed, which we attribute to the low in-plane anisotropy of the CuMnAs and its subsequent easy coherent rotation. This is supported by the XMLD data in the following sections. The rounded shape of the loop is ascribed to crystalline disorder, due to the large lattice mismatch between Fe and GaP (001).

### X-ray magnetic circular and linear dichroism measurements

The XMCD and XMLD measurements were performed on beamline I06-1 of Diamond Light Source, using total electron yield detection and a superconducting vector magnet in which magnetic fields can be applied in any direction. XMCD spectra were measured with the x-ray beam at a grazing angle of 25° to the sample surface, and with a magnetic field of 1000 Oe applied along the beam direction, as illustrated in Fig. 1d. Figure 1e and f show the Fe *L*_2,3_ and Mn *L*_2,3_ x-ray absorption and XMCD spectra from the sample, at a temperature of 250 K. The Mn XMCD is very weak and of opposite sign to the Fe XMCD, indicating a small net Mn magnetic moment which is antiferromagnetically coupled to the Fe layer. The antiparallel alignments of the Fe and CuMnAs magnetic moments is in contrast to Fe_1−*x*_Mn_*x*_ binary alloys, for which the Mn moment is small and parallel to the Fe^25^. The magnitude of the XMCD asymmetry (*I* ^+^ − *I* ^−^)/(*I* ^+^ + *I* ^−^), where *I* ^+^ and *I* ^−^ are the Mn *L*_3_ peak heights above background for photon helicity parallel and antiparallel to the magnetic field, is around 1%.

As shown in Fig. 1a, the magnetic structure in CuMnAs consists of FM (001) planes which are AF coupled to the neighbouring sublattice planes. Therefore, the interface plane of CuMnAs may be expected to consist of uncompensated Mn magnetic moments. Due to the finite probing depth of the total electron yield XMCD measurement, the signal from the uncompensated interface layer is not fully cancelled by the opposite oriented layer below it. The XMCD from the AF ordered CuMnAs film will be smaller than for a fully FM oriented CuMnAs film by a factor $R=(1-{e}^{-a/d})/(1+{e}^{-a/d})$, where *a* is the sublattice plane spacing and *d* is the total electron yield probing depth. Taking *d* ≈ 3 nm^26^ and *a* = 0.3 nm (ref. 2) gives *R* ≈ 0.05, consistent with the small size of the observed Mn XMCD. However, we do not rule out a possible contribution from rotatable uncompensated moments in the bulk of the AF layer, or interfacial alloying.

**Figure 2:**
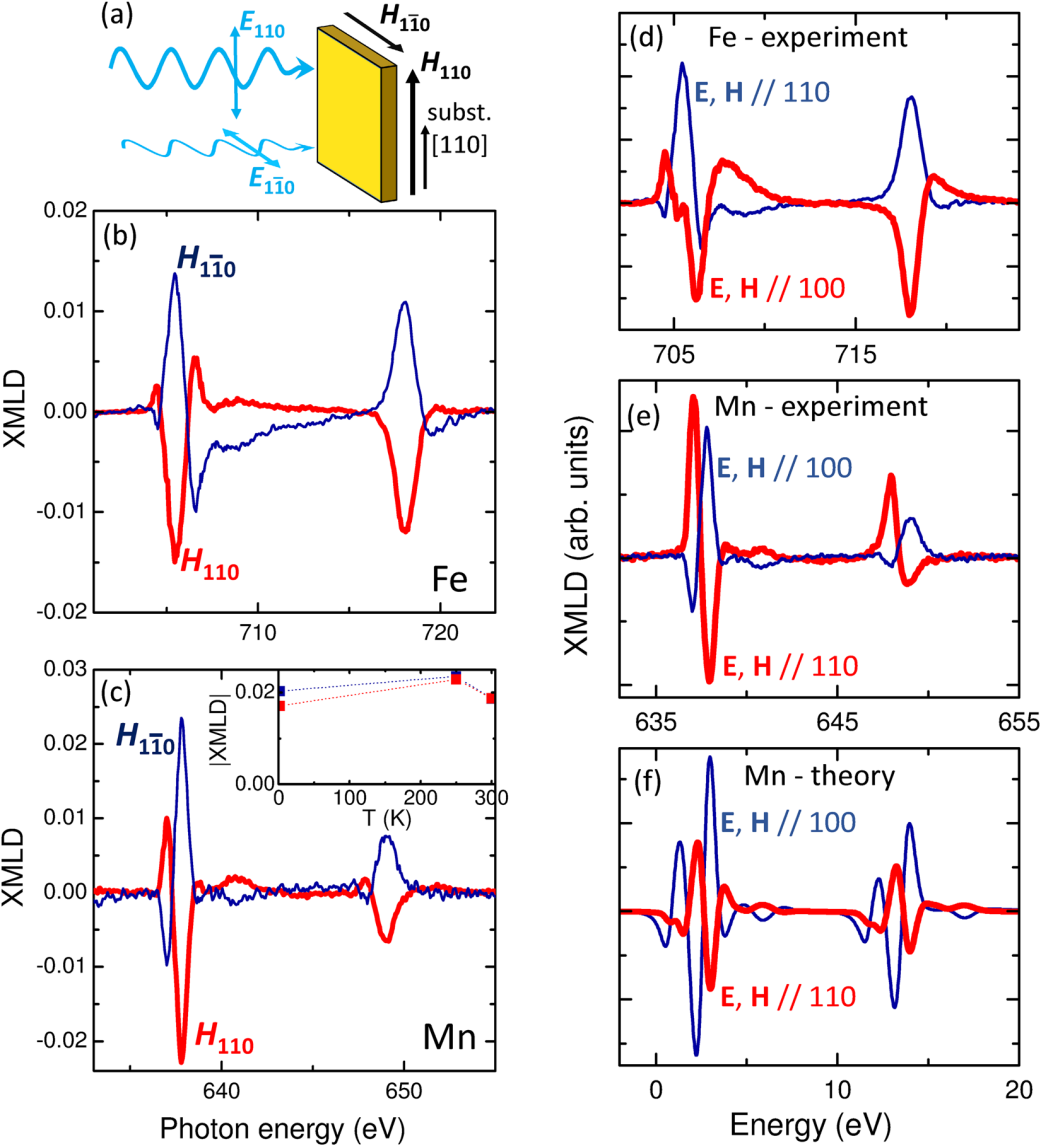
Rotation of the staggered AF moments due to exchange coupling, and anisotropic XMLD spectra. (**a**) Experimental geometry for the XMLD measurements. (**b**) Fe *L*_2,3_ and (**c**) Mn *L*_2,3_ XMLD spectra, obtained as the difference between absorption spectra measured with x-ray linear polarization vector along the [110] and $[1\bar{1}0]$ directions of the GaP substrate, with applied magnetic field along [110] (thick lines) and along $[1\bar{1}0]$ (thin lines). The inset to (**c**) shows the magnitude of the Mn *L*_3_ XMLD peak as a function of temperature. (**d**) Fe *L*_2,3_ and (**e**) Mn *L*_2,3_ anisotropic XMLD spectra, obtained from the difference between absorption spectra with parallel and perpendicular configurations of the x-ray polarization and the 1000 Oe applied magnetic field, for fields along 〈110〉 (thin blue lines) and 〈100〉 (thick red lines) in-plane axes. The experimental XMLD spectra in (**b**–**e**) are measured at temperature *T* = 250 K. (**f**) Calculated Mn *L*_2,3_ anisotropic XMLD spectra for tetragonal CuMnAs.

The XMLD spectra were obtained with the x-ray beam at normal incidence, taking the difference between absorption spectra measured with the x-ray linear polarization vector parallel to the [110] and $[1\bar{1}0]$ axes of the GaP substrate. A 1000 Oe magnetic field was applied along either the [110] or $[1\bar{1}0]$ axes, with a small out-of-plane tilt in order to increase the electron yield signal. It was verified that the small out-of-plane component of the field did not affect the spectra. The experimental geometry is illustrated in Fig. 2a. The XMLD spectra at the Mn *L*_2,3_ and Fe *L*_2,3_ edges at 250 K are shown in Fig. 2b and c respectively. The XMLD spectra are shown as a fraction of the *L*_3_ absorption peak height above background.

The Mn *L*_3_ XMLD signal is larger than that of the Fe and comparable to that of a 10 nm CuMnAs single layer^16^. Given the large size of the Mn XMLD signal, it can be inferred that it is due to the compensated antiferromagnetic Mn moments in the CuMnAs film rather than the small number of uncompensated moments at the interface. Most strikingly, the same XMLD signal, but with opposite sign, is observed when the applied magnetic field is applied in the orthogonal direction. The reversal of the XMLD spectrum is expected for the FM layer if the Fe magnetization orients parallel to the magnetic field. The observation of similar behaviour for the Mn XMLD indicates that the staggered magnetic moments in the CuMnAs layer have a uniaxial orientation and are exchange coupled to the Fe layer, following the reorientation of the Fe magnetization under the applied magnetic field. The rotation of the AF spins is also observed at 300 K and 2 K, although the magnitude of the XMLD is slightly reduced compared to its value at 250 K, as shown in the inset to Fig. 2c. The smaller value at 2 K may be due to competition between the interlayer exchange coupling and magnetocrystalline anisotropy in the CuMnAs layer.

Figure 2d and e compare XMLD spectra measured for x-ray polarization and applied magnetic fields along the in-plane [100] and [110] crystal axes. For both the Mn and Fe *L*_3_ absorption edges, the sign and lineshape of the XMLD depend strongly on the crystallographic direction. The Fe *L*_2,3_ XMLD spectra shown in Fig. 2d are in good agreement with previous studies of epitaxial Fe films on GaAs(001)^20^. This confirms that the Fe layer is epitaxial with in-plane crystal directions parallel to those of the substrate.

#### Electronic structure calculations

The Mn *L*_2,3_ XMLD spectra shown in Fig. 2e are compared to *ab initio* calculations shown in Fig. 2f. The theoretical XMLD spectra were obtained from LDA + U electronic structure calculations^2^ using the approach of ref. 13, which neglects electronic correlations and core hole effects. The finite core hole lifetime was mimicked by lorentzian broadening of 0.4 eV. The calculations reproduce some of the main features in the experimental spectra, including the relative separations of the main peaks and their reversal in sign between the different crystal orientations. Additional features on the low-energy side of the *L*_2_ and *L*_3_ peaks in the calculated spectra are not observed in the experiment. The experimental XMLD spectra are defined as the absorption spectra for parallel x-ray polarization and applied magnetic field, minus the absorption spectra for perpendicular x-ray polarization and applied magnetic field. Similarly, the calculated XMLD are the absorption for AF moments parallel to x-ray polarization, minus the absorption for AF moments perpendicular to polarization. Taking into account the 45° rotation of the CuMnAs crystal with respect to the GaP substrate^2^, the sign of the main peaks is in agreement between theory and experiment for both crystal orientations. The comparison of the measured spectra to the calculation therefore indicates that the AF spin axis in the CuMnAs layer is aligned collinear with the external magnetic field, *i*.*e*., the interlayer exchange coupling favours a collinear alignment of the FM Fe and AF CuMnAs magnetic moments.

## Discussion

From the XMCD and XMLD results described above, we can infer the following. The Mn XMCD is consistent with the interface atomic layer of the CuMnAs film orienting antiparallel to the epitaxial FM Fe layer as well as to the neighbouring CuMnAs magnetic sublattice, although other possible contributions to the measured XMCD signal (*e*.*g*. bulk uncoupled moments or interfacial alloying) cannot be ruled out. The AF CuMnAs spins have a collinear coupling to the Fe layer. The AF spins in the CuMnAs layer are rotatable by reorienting the Fe magnetization under relatively small external magnetic fields. This is in contrast to for example CoO/Fe epitaxial layers, where the AF spin configuration is largely frozen for thicknesses above ≈3 nm^13^. Interlayer exchange coupling therefore provides a means to rotate the orientation of compensated AF materials, which are hard to manipulate directly using external magnetic fields. For tetragonal CuMnAs, this may be combined with manipulation of the magnetic order using spin-orbit torques^9, 27^, and electrical^9^ or magneto-optical^11^ detection, for future hybrid FM/AF spintronic applications. Also significantly, the description of the XMLD lineshape in CuMnAs will allow for greater understanding of the domain structures imaged by XMLD. This is likely to become a field of great interest owing to the potential application of AF CuMnAs.
